# NSD2 and miRNAs as Key Regulators of Melanoma Response to Romidepsin and Interferon‐α2b Treatment

**DOI:** 10.1002/cam4.70917

**Published:** 2025-05-19

**Authors:** Alessandro De Santis, Lucrezia De Santis, Francesca Rossi, Silvia Gasparini, Valerio Licursi, Vito Antonio Amico, Imerio Capone, Alessandra Fragale, Stefania D'atri, Lucia Gabriele, Carlo Presutti

**Affiliations:** ^1^ Department of Biology and Biotechnology Charles Darwin Sapienza University of Rome Rome Italy; ^2^ Max Planck Institute for Molecular Genetics Chromatin Structure and Function Berlin Germany; ^3^ Institute of Molecular Biology and Pathology (IBPM) National Research Council (CNR) of Italy Rome Italy; ^4^ Molecular Oncology Laboratory Istituto Dermatopatico Dell'immacolata IDI‐IRCCS Rome Italy; ^5^ Istituto Superiore di Sanità Department of Oncology and Molecular Medicine Rome Italy

**Keywords:** epigenetic regulation, melanoma, microRNAs, NSD2, proliferative subtypes

## Abstract

**Background:**

We investigated the role of Nuclear Receptor Binding SET Domain Protein 2 (NSD2) and microRNAs (miRNAs) in melanoma de‐differentiation following Romidepsin and Interferon‐α2b (RI) treatment. Melanoma is the most lethal form of skin cancer, and despite advancements in therapy, treatment resistance remains a major challenge. De‐differentiation has been widely recognized as a key factor contributing to therapy resistance.

**Methods:**

RNA‐seq and TCGA transcriptomic data were re‐analyzed to identify miRNAs and NSD2 expressions. The functional impact of selected miRNAs was then investigated at the molecular and phenotypic levels using primary and immortalized cell lines.

**Results:**

Our findings demonstrate that RI treatment induces a de‐differentiation process in primary melanoma cells, resembling that observed in therapy‐resistant melanoma. This effect is particularly pronounced in cells with an intrinsic proliferative phenotype, where we observed significant downregulation of NSD2, a key epigenetic regulator implicated in multiple cancers. Additionally, we identified specific miRNAs as mediators of NSD2 downregulation, influencing melanoma cell viability and fitness.

**Conclusions:**

These findings provide new insights into the molecular mechanisms driving melanoma progression and highlight potential therapeutic targets to counteract treatment resistance.

## Graphical abstract

This figure illustrates the proposed mechanism of *NSD2* regulation in melanoma following romidepsin and interferon‐α2b (RI) treatment. RI induces a de‐differentiation process, particularly in proliferative melanoma cells, leading to *NSD2* downregulation. This effect is mediated by the upregulation of specific miRNAs (miR‐23a, miR‐24, and miR‐31), which target *NSD2* and influence downstream oncogenic pathways involving *CCND1*, *MYC*, and *BCL2*.

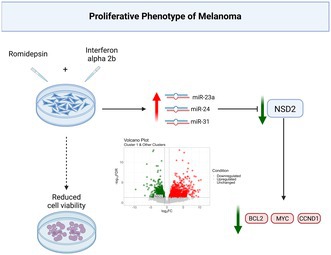

**Abbreviations**
3′ UTR: 3′ untranslated region
BRAFi: BRAF inhibition
EMT: epithelial‐mesenchymal transition
EC: endometrial cancer
GC: gastric cancer
GO: Gene Ontology
GSEA: Gene Set Enrichment Analysis
HDCAi: histone deacetylase inhibitor
H3K36me2: histone H3 lysine 36 di‐methylation
MAPK: mitogen‐activated protein kinase
MAPKi: pathway inhibition
miRNAs: microRNAs
NT: non‐treated
PCA: principal component analysis
RT‐qPCR: real‐time quantitative polymerase chain reaction
RTKs: receptor tyrosine kinases
RNAi: RNA interference
RNAseq: RNA sequencing
RI: romidepsin and interferon‐α2b
scRNA‐seq: single‐cell RNA sequencing
SCC: squamous cell carcinoma
VEM: vemurafenib

## 1 Introduction

Melanoma is a highly aggressive type of skin cancer that arises from melanocytes, the pigment‐producing cells located primarily in the epidermis. Due to its increasing incidence and poor prognosis in advanced stages, melanoma presents a significant challenge in oncology. It has seen significant advancements in treatment due to the discovery of B‐Raf Proto‐oncogene, serine/threonine kinase (*BRAF*) mutations driving half of all cutaneous melanomas and breakthroughs in tumor immunology. These discoveries led to targeted and immune therapies, resulting in remarkable response rates and improved survival for many patients [1]. Despite these successes, challenges remain, as some patients do not respond to therapies or eventually relapse [2, 3, 4]. One contributing factor to treatment resistance and disease progression is the phenotypic heterogeneity observed in melanoma cells [5, 6].

Researchers have uncovered compelling evidence indicating that melanoma cells can exhibit distinct transcriptional cell states, with some cells adopting a proliferative or melanocytic state, while others acquire a mesenchymal‐like or invasive state [7, 8]. Single‐cell RNA‐sequencing (scRNA‐Seq) analyses have confirmed the coexistence of these cell states in clinical samples, highlighting the complexity of intratumor heterogeneity in melanoma [6, 8, 9]. During malignant transformation, melanoma cells may resemble distinct stages of embryonic development. Gene expression analyses of human melanoma cell lines and patient tumors have unveiled a two‐dimensional differentiation trajectory, further subdivided into four progressive subtypes: undifferentiated, neural crest‐like, transitory, and melanocytic [10]. These subtypes align with the well‐established Hoek classification, which refines the proliferative and invasive melanoma subtypes [11]. Specifically, the melanocytic and transitory phenotypes correspond to the proliferative subtype, while the undifferentiated and neural crest‐like phenotypes align with the invasive subtype. This classification further refines the traditional proliferative and invasive categories by introducing two additional subgroups, establishing a four‐stage stepwise differentiation model based on normal melanocyte differentiation programs. These subgroups possess varying fitness levels, allowing some to outcompete others. Specific markers are associated with these signatures such as transcription factors (TFs) and receptor tyrosine kinases (RTKs). Markers of proliferative states—such as the melanocytic and transitory states—include melanocyte‐inducing transcription factor (*MITF*), melan‐A (*MLANA*), and SRY‐box transcription factor 10 (*SOX10*) [10, 12] In contrast, markers of invasive states, such as the neural crest‐like and undifferentiated phenotypes, include AXL receptor tyrosine kinase (*AXL*), SRY‐box transcription factor 9 (*SOX9*), and Epidermal growth factor receptor (*EGFR*) [13].

Resistance to BRAF inhibition (BRAFi) is a significant clinical obstacle, as certain melanoma cells can downregulate key regulators of melanocyte differentiation, such as *MITF*, and upregulate RTKs like *AXL* and *EGFR* [14, 15]. These molecular changes are linked to intrinsic resistance to mitogen‐activated protein kinase (*MAPK*) inhibitors (MAPKi) and are commonly observed in patient tumors during disease progression on MAPKi therapies [6, 16, 17]. Furthermore, melanoma cells often undergo de‐differentiation upon acquiring resistance to MAPKi, evidenced by shifts toward an undifferentiated phenotype in certain melanoma cell lines [10]. Despite advancements, 15%–20% of tumors exhibit primary resistance to current therapies, and acquired resistance remains a critical challenge. As a result, the urgency to develop novel therapeutic approaches for melanoma is evident.

One promising combination therapy for melanoma involves romidepsin and interferon α2b (RI) [18]. Romidepsin, an FDA‐approved drug with multiple cancer indications, acts as an inhibitor of Class I histone deacetylases (HDAC) [19], while interferon α2b was among the first drugs developed specifically for melanoma treatment, activating a transcriptional program that exhibits anti‐cancer activity [20]. However, the clinical usage of interferon α2b has been limited due to the requirement for high doses of the drug, which can lead to undesirable side effects. The combination of Romidepsin and interferon α2b presents a promising strategy to address this challenge. Romidepsin's epigenetic activity enables it to open chromatin, enhancing accessibility to target sites, while interferon α2b can be more effectively utilized at lower doses within this combination [21]. In our previous study [18], this drug combination was shown to reduce melanoma proliferation, enhance long‐term survival, suppress invasiveness, and overcome acquired resistance to vemurafenib (VEM). In the present study, we re‐analyze the transcriptomic effects of RI treatment on primary melanoma cell lines to further investigate its molecular impact on de‐differentiation and therapy resistance.

*NSD2*, also known as *WHSC1*, catalyzes histone H3 lysine 36 di‐methylation (H3K36me2) and is implicated in tumorigenesis, DNA damage repair, and epithelial‐mesenchymal transition (EMT) processes [22, 23, 24, 25]. *NSD2* has been shown to play a role in cancer progression by regulating key oncogenes, such as cyclin D1 (*CCND1*), MYC proto‐oncogene, BHLH transcription factor (*MYC*), and BCL2 apoptosis regulator (*BCL*2), which contribute to cell survival and proliferation [26, 27, 28, 29, 30, 31, 32]. While *NSD2* is a well‐established oncogene, its role in melanoma has not been previously characterized. Additionally, *NSD2* expression is post‐transcriptionally regulated by several miRNAs, which control its levels by targeting its 3′ untranslated region (3′ UTR) [33, 34, 35, 36]. MiRNAs themselves can play crucial roles in melanoma and other cancers, including miR‐23a, miR‐24, and miR‐31. These miRNAs are downregulated in melanoma and their expression correlates with poor outcome [37, 38, 39, 40, 41].

This study aims to explore the role of *NSD2* and miRNAs in melanoma's response to the RI. We hypothesize that specific miRNAs may regulate *NSD2* expression, and in turn, influence the cellular response to this therapy. By understanding these molecular interactions, we hope to identify novel therapeutic strategies to enhance treatment efficacy and overcome resistance in melanoma.

## 2 Results

### 2.1 RI Treatment Induces a de‐Differentiation Process

**FIGURE 1:**
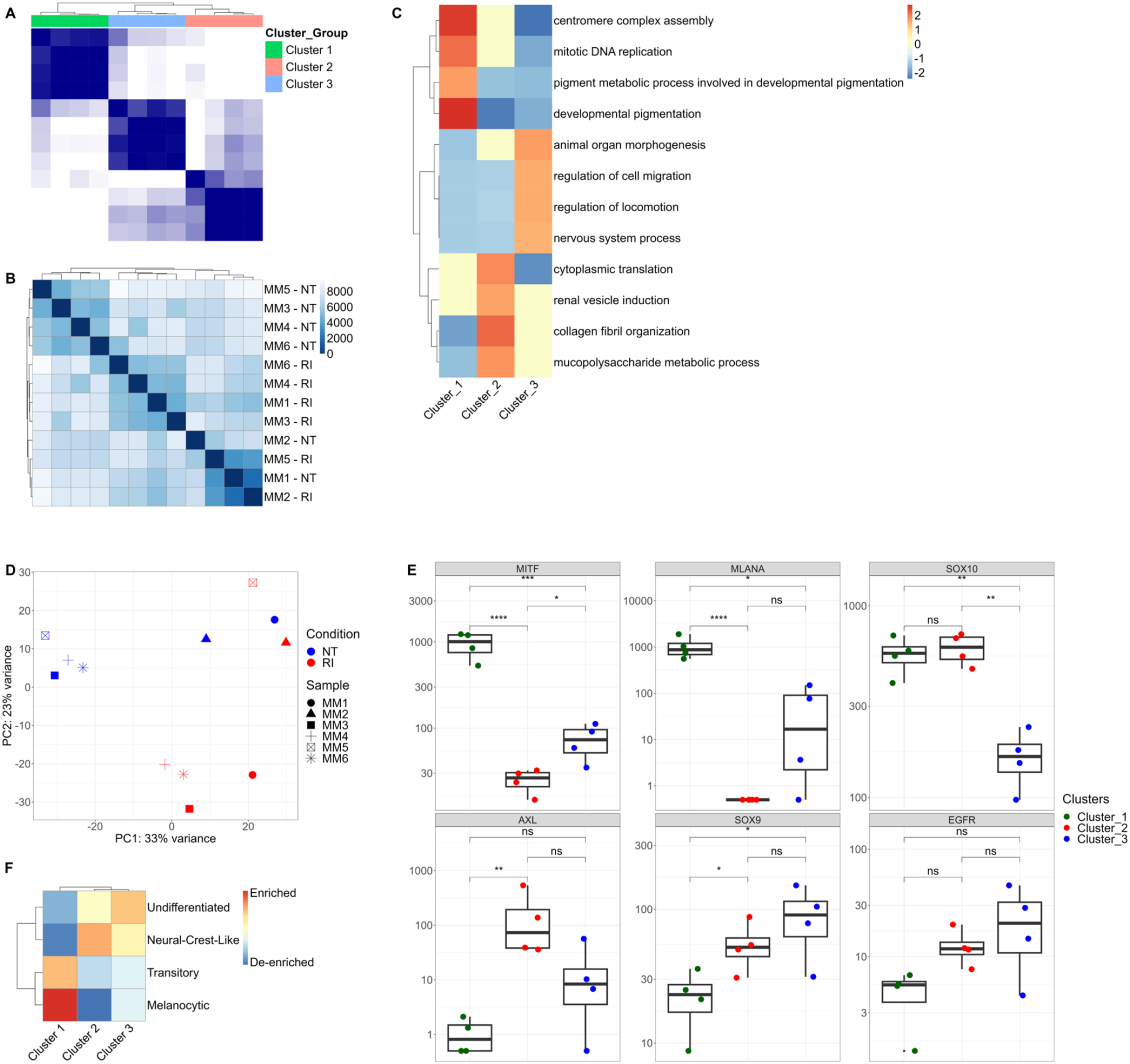
RNA‐Seq Analysis. Data sourced from the GSE221386 dataset. (A) Consensus indices matrix resulting from hierarchical clustering of primary melanoma cell lines, grouped by setting *k* = 3 clusters. (B) Heatmap displaying sample‐to‐sample distances, calculated from the variance‐stabilizing transformation of count data for overall gene expression. (C) Summary heatmap of enrichment analysis *p*‐values for each individual cluster compared to the remaining clusters. (D) Principal component analysis (PCA) plot of melanoma cell lines. Each color represents the treatment condition, and shapes represent different cell lines. (E) Boxplots depicting the expression levels of select transcription factors and receptor tyrosine kinase (RTK) genes. Boxplot lines indicate the lower quartile, median, and upper quartile, with whiskers reflecting 1.5 times above or below the interquartile range. (*p*‐values: ns > 0.05, * ≤ 0.05, ** ≤ 0.01, *** ≤ 0.001).

Six primary melanoma cell lines (MM1–MM6) were treated with RI, as previously reported in Fragale et al. [18] We re‐analyzed the transcriptomic data from these treated and untreated samples using RNA sequencing (RNA‐Seq) to investigate differential gene expression and clustering patterns. Consensus hierarchical clustering was performed on RI‐treated samples, as this combination induced the most significant reduction in cell viability. The analysis aimed to assess sample similarity and clustering patterns. The results revealed three distinct clusters among the 12 samples (untreated, NT, and RI‐treated) (Figure 1A,B). Cluster 1 consisted of all untreated cell lines (MM3‐NT, MM4‐NT, MM5‐NT, and MM6‐NT). Cluster 2 included a mix of untreated and treated samples (MM1‐NT, MM2‐NT, MM2‐RI, MM5‐RI), while Cluster 3 contained only RI‐treated cells (MM1‐RI, MM3‐RI, MM4‐RI, MM6‐RI). Gene set enrichment analysis (GSEA) identified biological processes associated with proliferation and melanocytic differentiation in Cluster 1, whereas Cluster 3 exhibited enrichment for de‐differentiation and invasion pathways (Figure 1C). Cluster 2 represented an intermediate transcriptomic state. Principal component analysis (PCA) confirmed a clear separation between the three clusters (Figure 1D). Analysis of key melanoma signature markers (Figure 1E) revealed that *MITF* and *MLANA* were highly expressed in Cluster 1, indicating a differentiated state. *SOX10* showed significant expression in Clusters 1 and 2, while *SOX9*, *AXL*, and *EGFR* were enriched in Clusters 2 and 3, suggesting a more undifferentiated and invasive phenotype. Notably, *AXL* exhibited significant enrichment in Cluster 2, reinforcing its association with invasive melanoma subtypes.

Enrichment analysis of melanoma transcriptomic subtypes (Figure 1F) showed that Cluster 1 correlated with a melanocytic/transitory signature, while Clusters 2 and 3 were enriched for a neural crest‐like/undifferentiated phenotype. These findings suggest that the untreated primary cell lines exhibit different initial differentiation states and that RI treatment induces a de‐differentiation process, similar to that observed with vemurafenib resistance. Based on these findings, we designated the untreated cell lines in Cluster 1 (MM3, MM4, MM5, MM6) as “Proliferative” and those in Cluster 2 (MM1, MM2) as “Invasive.”

### 2.2 miRNA Regulation of NSD2 in Melanoma Subtypes

**FIGURE 2:**
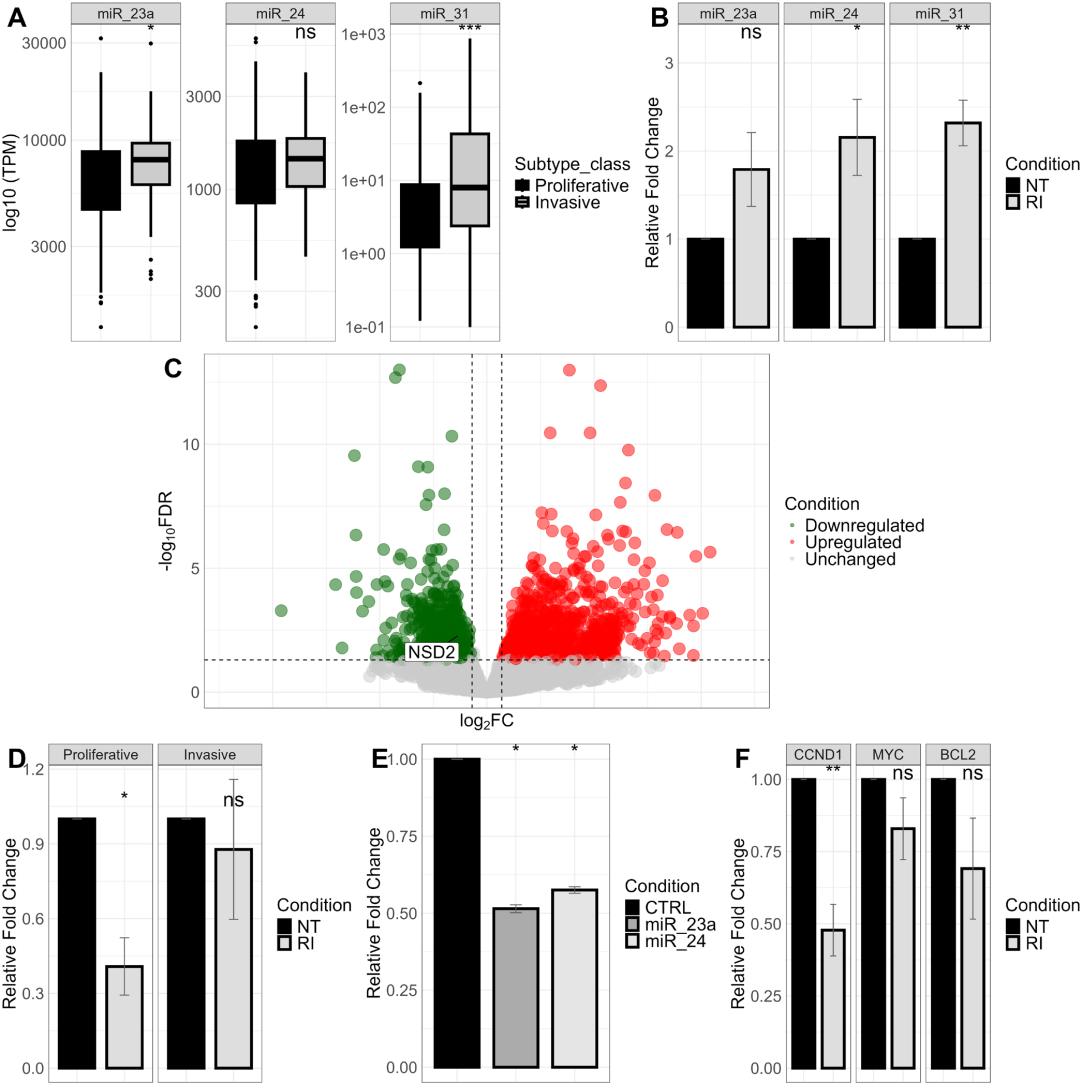
Analysis of miRNA‐mediated regulation of NSD2 in melanoma subtypes. (A) Comparative analysis of TCGA melanoma miRome datasets identifying differentially expressed miRNAs in invasive melanoma subtypes. (B) RT‐qPCR analysis of miRNAs following treatment with RI in the primary proliferative cell lines. The y‐axis represents the relative fold change compared to the control condition. Each color represents a different treatment condition. Mean and standard error (SE) are depicted in the bar chart. (*p*‐values: ns > 0.05, * ≤ 0.05, ** ≤ 0.01, *** ≤ 0.001). (C) Volcano plot illustrating modulated genes in primary melanoma cells of Cluster 1 cells versus Cluster 2, 3, displaying each gene's − log10 (FDR) and log2 fold change with the chosen covariate. Upregulated genes (FDR ≤ 0.1 and log2 fold change > 0.7) are highlighted in red. Downregulated genes (FDR ≤ 0.1 and log2 fold change < 0.7) are indicated in green. Vertical dashed lines indicate thresholds for up‐regulated and down‐regulated genes, and the horizontal dashed line represents the FDR threshold. (D) Real‐time quantitative polymerase chain reaction (RT‐qPCR) analysis of NSD2 following treatment with RI in the proliferative and invasive cell lines. The y‐axis represents the relative fold change compared to the control condition. Each color represents a different treatment condition. Mean and standard error (SE) are depicted in the bar chart. (*p*‐values: ns > 0.05, * ≤ 0.05, ** ≤ 0.01, *** ≤ 0.001). (E) Dual luciferase reporter assays demonstrating miRNA‐mediated target repression. Results are presented as the ratio of luciferase activity of miRNA/negative control normalized to empty vector control. Data represent three independent experiments and are presented as mean ± SE. (F) RT‐qPCR analysis of NSD2's targets (CCND1, MYC, BCL2) following treatment with RI. The y‐axis represents the relative fold change compared to the control condition. Each color represents a different treatment condition. Mean and standard error (SE) are depicted in the bar chart. (*p*‐values: ns > 0.05, * ≤ 0.05, ** ≤ 0.01, *** ≤ 0.001).

To explore the role of miRNAs in melanoma subtypes, we analyzed TCGA melanoma miRome datasets comprehending 452 patients' biopsies, stratified by transcriptomic subtype according to the Hoek classification. Comparative analysis identified miRNAs overexpressed in invasive subtypes, leading to the selection of three candidates: miR‐23a, miR‐24, and miR‐31 (Figure 2A). We validated the upregulation of these miRNAs in our primary melanoma cell lines following RI treatment (Figure 2B). We utilized TargetScan, miRTarBase, and Minturnet to identify their potential gene targets, cross‐referencing the predicted targets with RNA‐Seq data from RI‐treated primary cells. *NSD2* emerged as a target of all three miRNAs and was found downregulated in the invasive clusters (Clusters 2 and 3) compared to the proliferative cluster (Cluster 1) (Figure 2C). RT‐qPCR analysis confirmed that *NSD2* expression was significantly downregulated after RI treatment, specifically in proliferative melanoma cell lines (Figure 2D). This suggests that *NSD2* downregulation in response to RI is dependent on the initial transcriptomic state of the cells.

To validate *NSD2* as a direct target of miR‐23a and miR‐24, we performed a luciferase reporter assay containing the full‐length 3′‐UTR of *NSD2*. Transient transduction of HeLa cells with miR‐23a or miR‐24 resulted in a ~45% reduction in luciferase activity, confirming the specificity of the interaction (Figure 2E). MiR‐31 had already been validated as an *NSD2* regulator in previous studies. *NSD2* plays a crucial role in epigenetic regulation and transcriptional control. We investigated three key *NSD2*‐regulated oncogenes: *CCND1*, *MYC*, and *BCL2*. RI treatment led to the downregulation of *CCND1*, *MYC*, and *BCL2* in proliferative cell lines, further supporting the role of *NSD2* in melanoma progression (Figure 2F).

### 2.3 Impact of miRNA Overexpression and siNSD2 on Melanoma Cell Behavior

**FIGURE 3:**
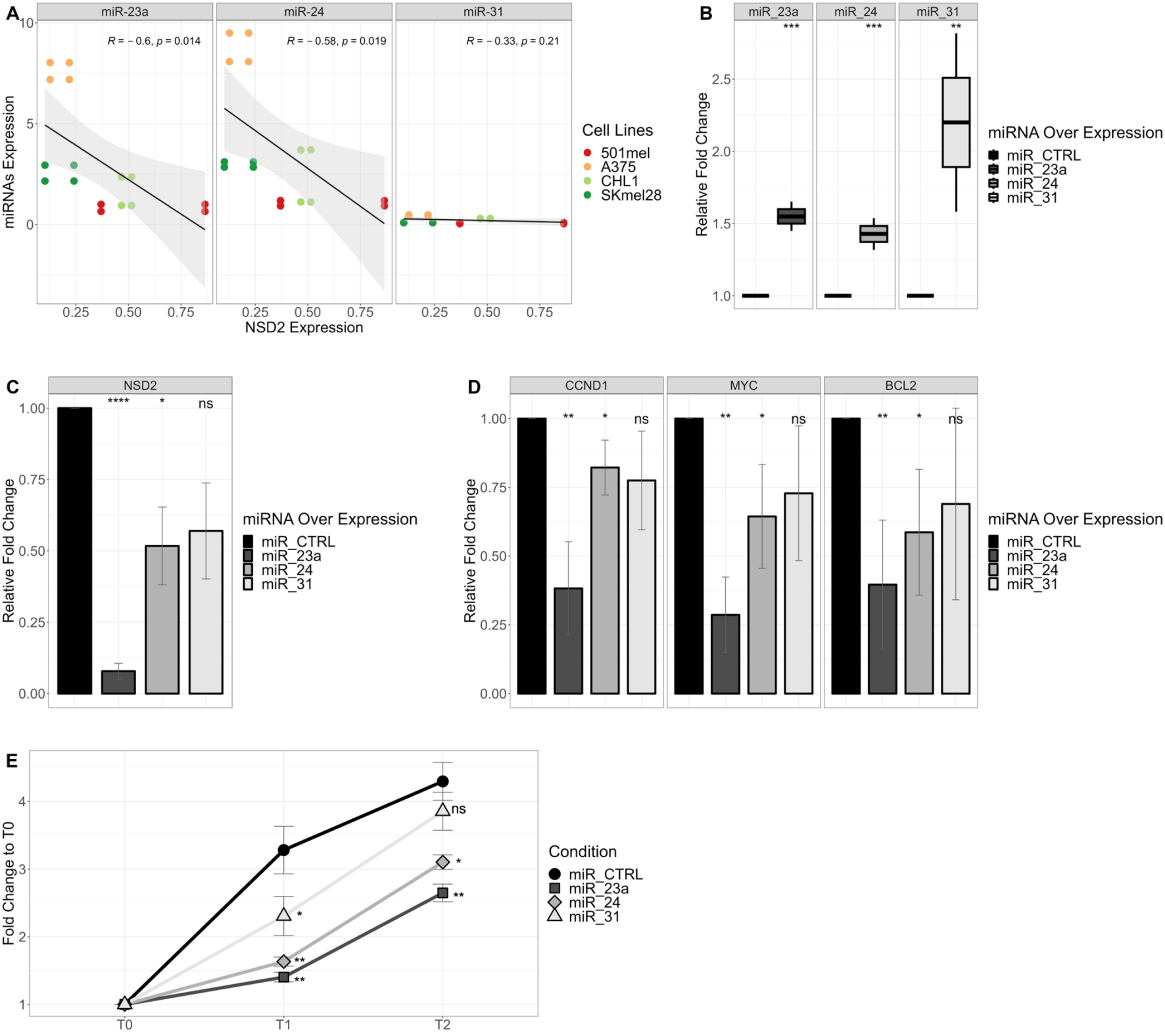
miRNA overexpression and correlation with NSD2. (A) Correlation of expression between miRNAs and NSD2 in human melanoma cells. NSD2 and miRNA expression were calculated with RT‐qPCR, and the 2^−ΔCT^ values were plotted on the x and y axes, respectively. Each color represents a different cell line. Pearson correlation coefficient is depicted as R and the *p*‐value is *p*. (B) Validation of miRNA overexpression through RT‐qPCR. The y‐axis represents the relative fold change compared to the control condition. Each color represents a different miRNA/CTRL overexpression. Mean and standard error (SE) are depicted in the bar chart. (*p*‐values: ns > 0.05, * ≤ 0.05, ** ≤ 0.01, *** ≤ 0.001). (C‐D) RT‐qPCR of NSD2 and NSD2's targets after miRNA overexpression. (E) Proliferation assay shows relative growth inhibition compared to T0. Each color represents a different miRNA/miCTRL overexpression. (T0 = 0 h, T1 = 24 h, T2 = 48 h after transfection).

To explore the impact of three miRNAs in human melanoma cells, we initially examined their expression correlation with *NSD2* across four human cell lines: SKmel28, 501mel, A375, and CHL1. Notably, miR‐23a and miR‐24 exhibited a significant anti‐correlation with *NSD2* expression, while miR‐31 demonstrated a non‐significant anti‐correlation, possibly due to its low expression in human melanoma cells (Figure 3A). Subsequently, we transiently overexpressed each miRNA in SKmel28 cells and confirmed the overexpression through RT‐qPCR analysis (Figure 3B). We then validated the downregulation of *NSD2* following miRNA overexpression (Figure 3C). MiR‐23a demonstrated a substantial reduction of *NSD2* messenger levels, with a 90% decrease in expression, while miR‐24 and miR‐31 showed approximately 50% reduction. Furthermore, we assessed the expression of *CCND1*, *MYC*, and *BCL2* via RT‐qPCR (Figure 3D). We observed significant downregulation of all three targets with miR‐23a overexpression. In contrast, miR‐24 and miR‐31 displayed varied effects on the expression of these targets. Finally, we evaluated the impact of ectopic miRNA overexpression on the proliferation of human melanoma cells (Figure 3E). Our results indicated a greater reduction in proliferation with miR‐23a overexpression, followed by miR‐24 and miR‐31.

**FIGURE 4:**
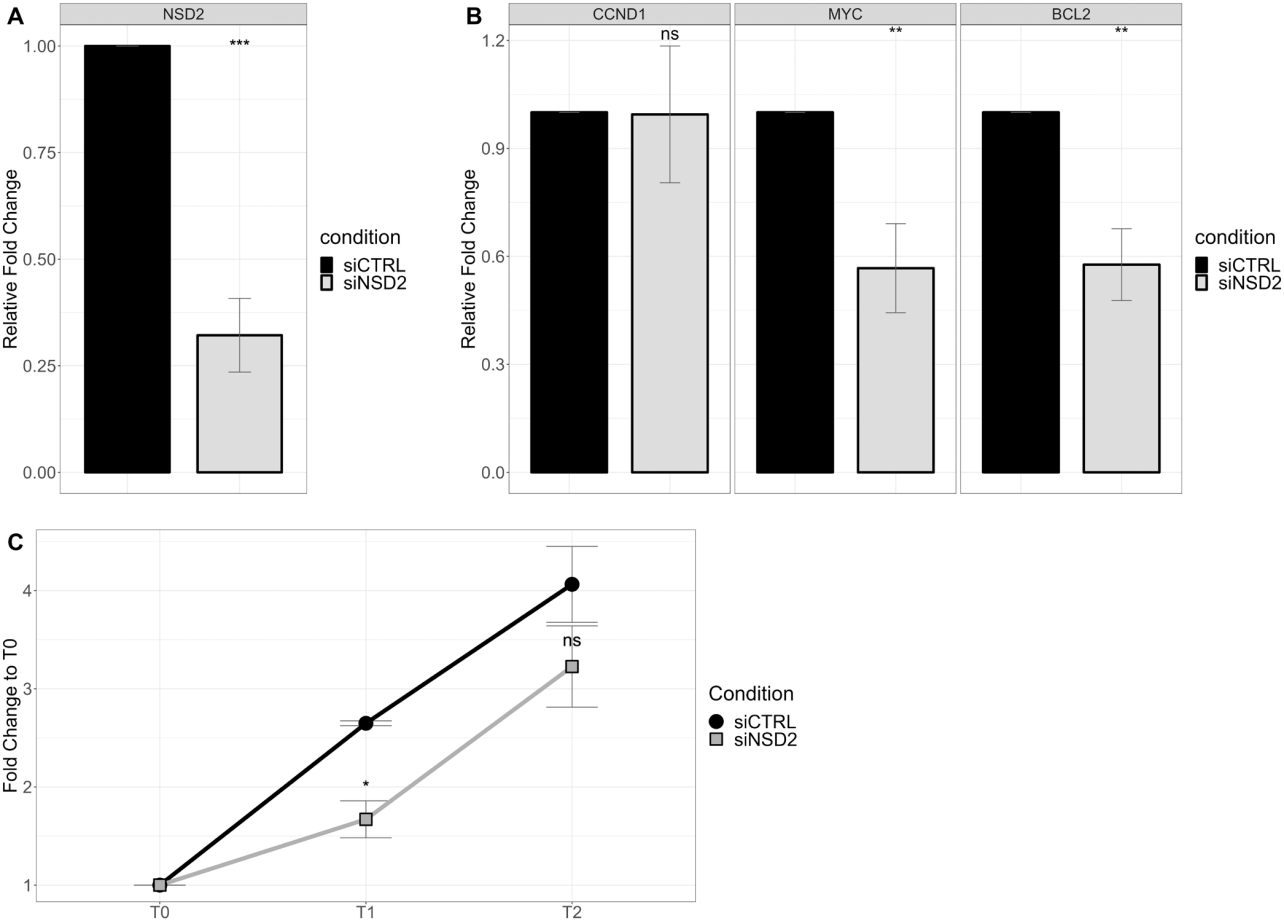
siNSD2. (A) Validation of NSD2 silencing with RT‐qPCR. The y‐axis represents the relative fold change compared to the control condition. Each color represents a different siRNA condition. Mean and standard error (SE) are depicted in the bar chart. (*p*‐values: ns > 0.05, * ≤ 0.05, ** ≤ 0.01, *** ≤ 0.001). (B) Downregulation of NSD2's targets after silencing of NSD2 is measured with RT‐qPCR. (C) Proliferation assay shows relative growth inhibition compared to T0. Each color represents a different siRNA condition. (T0 = 0 h, T1 = 24 h, T2 = 48 h after transfection).

RNA interference (RNAi) targeting *NSD2* was employed to assess whether the observed molecular effects on *NSD2* targets directly contributed to the reduction in proliferation or were the result of a pleiotropic effect of miRNAs. As depicted in Figure 4A, efficient downregulation of *NSD2* at the messenger level was achieved following *NSD2* silencing. Subsequently, the impact on *NSD2* targets, namely *MYC* and *BCL2*, was evident, whereas no significant downregulation was observed for *CCND1* (Figure 4B). Interestingly, the effect on proliferation was only apparent at time point T1, with no significant reduction observed at other time points (Figure 4C). Our study offers insights into the molecular dynamics governing melanoma cell competition, particularly in response to RI treatment. By identifying *NSD2* and miRNAs as potential mediators of the treatment response, we provide potential targets for intervention on the landscape of cells.

## 3 Discussion

The complex nature of melanoma, characterized by its diverse phenotypic variations, presents a significant challenge in the development of effective therapies. Resistance to current frontline treatments, such as *BRAF* inhibitors (BRAFi), remains a major obstacle in melanoma treatment. Phenotypic heterogeneity is a key factor in this resistance, with melanoma cells broadly classified into two predominant transcriptional states: the proliferative and invasive phenotypes. Recent studies have further refined this classification into a four‐stage de‐differentiation process, highlighting the dynamic plasticity of melanoma cells.

In this context, our findings indicate that RI treatment induces a de‐differentiation process in primary melanoma cells, resembling that observed in therapy‐resistant states. This process appears to be influenced by the initial transcriptomic state of the cells, with proliferative and more differentiated cells undergoing the most significant transcriptional changes. We identified *NSD2* as a key regulator of the proliferative phenotype, with its downregulation following RI treatment correlating with cellular de‐differentiation. Furthermore, our study highlights the role of three specific miRNAs (miR‐23a, miR‐24, and miR‐31) as mediators of *NSD2* downregulation, suggesting a regulatory mechanism underlying melanoma cell plasticity.

Given the critical role of *NSD2* in melanoma progression, therapeutic strategies targeting *NSD2* expression or activity—including miRNA‐based therapies or small‐molecule inhibitors—may offer new avenues for improving treatment outcomes. Our study provides novel insights into the molecular mechanisms governing melanoma cell competition, particularly in response to RI treatment, and identifies potential molecular targets for therapeutic intervention in melanoma.

## 4 Materials and Methods

### 4.1 RNA‐Seq Analysis

RNA sequencing of RI‐treated and untreated primary melanoma cell lines was previously performed and described in our previous work [18]. In the present study, we re‐analyzed these RNAseq data to assess differential gene expression and clustering patterns. Raw sequencing reads were obtained from the study and processed using TrimGalore for adapter trimming, followed by alignment to the human reference genome (GRCh38) using STAR aligner under default settings. Quality control was conducted using FastQC, and transcript abundance was quantified using HTSeq‐count version 0.8.0 script. Differential gene expression (DEG) analysis was conducted with DESeq2 (log2 fold change ≥ 1, adjusted *p*‐value < 0.05). GSEA was performed with the BROAD Institute's Molecular Signatures Database (MSigDB v7.4.1). Consensus clustering was performed using the ConsensusClusterPlus R package (version 1.64.0) on variance‐stabilized RNA sequencing data. Raw count data were normalized using DESeq2 and transformed with regularized log transformation (rlog) to stabilize variance across samples. The transformed expression matrix was used as input for clustering. The top 2000 most variable genes were selected based on variance across samples. Clustering was performed using hierarchical clustering with Euclidean distance. Genes associated with differentiation clusters were sourced from the study by Tsoi et al. [10] Graphs were made using ggplot2 DESeq2 version 3.5.1. TCGA TPM of miRNA was used to retrieve the expression.

### 4.2 Real‐Time qPCR

Total RNA was extracted using the miRNeasy Mini Kit (QIAGEN) and reverse‐transcribed with the High‐Capacity cDNA Reverse Transcription Kit (Applied Biosystems). Quantitative PCR (qPCR) was performed using Sensitive SYBR Green (Meridian Bioscience) on a StepOnePlus system (Applied Biosystems). Reactions were run in triplicates under the following conditions: initial denaturation at 95°C for 10 min, followed by 40 cycles of 95°C for 15 s and 60°C for 1 min. Melt curve analysis was performed to confirm specificity. Gene expression was normalized using HPRT (for mRNAs) and U6 (for miRNAs), and relative expression was calculated using the 2^(−ΔΔCt)^ method.

### 4.3 Cell Culturing

Primary melanoma cells were isolated from metastatic melanoma patient biopsies. Treatment with RI was performed as previously described [18]. Cells were cultured in RPMI 1640 medium (Euroclone SpA) supplemented with 10% FBS, 2 mM Glutamax, and 1% Pen/Strep. SKmel28 cells (ATCC) were cultured under the same conditions. All cell lines were routinely tested for mycoplasma contamination using the MycoAlert Mycoplasma Detection Kit (Lonza).

### 4.4 Cloning and Overexpression Assay

MiR‐23a, miR‐24, and miR‐31 were cloned into the PsiUX plasmid using XhoI and BglII restriction enzymes (New England Biolabs). Cloned constructs were sequence‐verified (Sanger sequencing). The full‐length 3′ UTR of NSD2 was cloned in psiCHECK‐2 vector that was digested with XhoI and NotI using InFusion method (Takara Bio) following the manufacturer's protocols. MiRNA overexpression was performed using Lipofectamine 3000 (Invitrogen) at a ratio of 2.5 μg plasmid per 6‐well plate. Cells were incubated for 48 h before RNA extraction. A scramble miRNA plasmid was used as a negative control.

### 4.5 Luciferase Assay

For the dual luciferase reporter assay, psiCHECK‐2 3′UTR constructs were co‐transfected with miRNA plasmids using Lipofectamine 3000 (Invitrogen). After 48 h, firefly and Renilla luciferase activities were measured using the Dual‐Glo Luciferase Assay System (Promega). Firefly luciferase activity was normalized to Renilla luciferase, and results were expressed as fold change relative to control.

### 4.6 Proliferation Assay

Cell viability was assessed using the Cell Counting Kit‐8 (CCK8, Sigma‐Aldrich). About 5000 cells per well were seeded in a 96‐well plate and treated with miRNA overexpression or siRNA knockdown. Absorbance was measured at 450 nm using a SpectraMax iD3 Microplate Reader (Molecular Devices) at 24 h, 48 h, and 72 h post‐treatment.

## Author Contributions

A.D.S., C.P., and L.G. conceptualized the study. A.D.S., F.R., L.G., and C.P. contributed to the study design. A.D.S., C.P., and L.G. wrote the original draft. A.D.S., L.D.S., L.G., and C.P. reviewed and edited the manuscript and approved the final version. A.D.S. generated visualizations. A.D.S., L.D.S., F.R., S.G., and V.A.A. conducted the experiments. A.D.S. and V.L. performed the bioinformatics analysis. I.C., A.F., and S.D. collected primary cells and performed RI treatment. The study was conducted under the supervision of L.G. and C.P. All authors had access to the study data and approved the final submission.

## Conflicts of Interest

The authors declare no conflicts of interest.

## Data Availability

Data sharing is not applicable to this article as no new data were created or analyzed in this study.
